# Adverse outcomes in maternity care for women with a low risk profile in The Netherlands: a case series analysis

**DOI:** 10.1186/1471-2393-13-219

**Published:** 2013-11-29

**Authors:** Lucie Martijn, Annelies Jacobs, Marianne Amelink-Verburg, Renske Wentzel, Simone Buitendijk, Michel Wensing

**Affiliations:** 1 IQ healthcare, Scientific Institute for Quality of Healthcare, Radboud University Nijmegen Medical Centre, 114 IQ healthcare, P.O. Box 9101, 6500, HB Nijmegen, The Netherlands; 2 Dutch Health Care Inspectorate, The Hague, The Netherlands; 3 Leiden University Medical Center, Leiden, The Netherlands

**Keywords:** Critical incidents, Primary midwifery care, Patient safety, Low risk pregnancy, Determinants of risk

## Abstract

**Background:**

This study aimed to perform a structural analysis of determinants of risk of critical incidents in care for women with a low risk profile at the start of pregnancy with a view on improving patient safety.

**Methods:**

We included 71 critical incidents in primary midwifery care and subsequent hospital care in case of referral after 36 weeks of pregnancy that were related to substandard care and for that reason were reported to the Health Care Inspectorate in The Netherlands in 36 months (n = 357). We performed a case-by-case analysis, using a previously validated instrument which covered five broad domains: healthcare organization, communication between healthcare providers, patient risk factors, clinical management, and clinical outcomes.

**Results:**

Determinants that were associated with risk concerned healthcare organization (n = 20 incidents), communication about treatment procedures (n = 39), referral processes (n = 19), risk assessment by telephone triage (n = 10), and clinical management in an out of hours setting (n = 19). The 71 critical incidents included three cases of maternal death, eight cases of severe maternal morbidity, 42 perinatal deaths and 12 critical incidents with severe morbidity for the child. Suboptimal prenatal risk assessment, a delay in availability of health care providers in urgent situations, miscommunication about treatment between care providers, and miscommunication with patients in situations with a language barrier were associated with safety risks.

**Conclusions:**

Systematic analysis of critical incidents improves insight in determinants of safety risk. The wide variety of determinants of risk of critical incidents implies that there is no single intervention to improve patient safety in the care for pregnant women with initially a low risk profile.

## Background

In many parts of the world, maternity care is provided in a multi-disciplinary team or network involving general physicians, obstetric specialists and midwives [1]. In The Netherlands, the start of maternity care is often provided in primary care practices [2]. Midwives refer a pregnant woman to an obstetric department in a hospital when an increased risk of complications is expected. Recent figures show that 80% of all the pregnant women in The Netherlands have a low risk pregnancy profile in early pregnancy and receive primary midwifery care, about 30% of these a priori low risk pregnant women are being referred to a hospital mainly during the third trimester of their pregnancy, and 20% of these women are referred while giving birth [3]. The remaining 30% of the low risk pregnant women remain in primary midwifery care and give birth, either at home (18%) or in a hospital (12%).

Perinatal mortality is showing a downward trend in The Netherlands, but other European countries have reported a more impressive decline in the mortality rates [4,5]. Although the impact of the Dutch perinatal system, as described above, is difficult to substantiate, one study has reported on adverse effects of this system on perinatal outcomes [6]. On the other hand, a large national study found no relation between births led by primary care midwives and increased risk of perinatal death in The Netherlands [7]. A study on maternal outcomes among low risk women with planned home versus hospital births in The Netherlands also showed that low risk women in primary care at the onset of labour with planned home birth had lower rates of severe acute maternal morbidity than those with planned hospital birth [8].

Several countries are developing policies to strengthen primary care for pregnant women. For instance, the recent ‘Birthplace in England national prospective cohort study’ supports a policy of offering healthy women with low risk pregnancies a choice of birth setting. All women planning birth at home or in a midwifery led care unit receive fewer interventions than those planning birth in an obstetric unit. There is no impact on perinatal outcomes for women planning birth at home or in a midwifery unit compared to women planning birth in an obstetric unit, except for primiparous women planning birth at home where there is an increase in adverse perinatal outcomes [9]. A Dutch patient record study of patient safety incidents in primary midwifery care showed that incidents in care provided by midwives do occur, but no safety incidents were associated with mortality or permanent harm [10]. The first results of the Dutch perinatal audit, a continuous monitoring of perinatal mortality after 37 weeks of pregnancy in The Netherlands, showed that in 10% of the evaluated cases, care was not provided in accordance with prevailing clinical guidelines and good clinical practice, and was defined as ‘substandard care’ [11].

Most studies on perinatal care focus on outcomes such as morbidity and mortality but do not provide information about underlying causes and effects. A case-by-case analysis of care for pregnant women with adverse outcomes provides information on determinants of safety risks. The database of the Dutch Health Care Inspectorate (DHI) contains these cases with care related unexpected untoward outcomes and is therefore a valuable source for analysis of critical incidents. Given the high number of referrals from pregnant women to hospital care in the third trimester or during birth, and the low a priori chance of adverse outcomes in this population, the challenge is to identify risk domains in the care for this majority of pregnant women in The Netherlands regardless of the echelon where this is provided. We focused our analysis on primary midwifery care (and additional primary care providers) for low risk pregnant women and hospital care for these women in case of referral after 36 weeks of pregnancy. The DHI database does not reflect the population at large, but contains cases that were reported by care providers and according to a DHI analysis, are related to a substandard quality of care. In this study we reviewed the critical incidents and final assessment by the DHI in care for women with a low risk pregnancy profile and aimed to analyse main determinants of risk.

## Methods

### Context

The Dutch Health Care inspectorate has an independent responsibility for supervision of quality and safety in healthcare. The supervision performed by the DHI is based on legislation and regulations as well as on ‘field standards’ set by professional associations. A significant approach for supervision is the evaluation of critical incidents in hospitals or primary care practices [12]. Under the Dutch Quality Act of 2005, health care professionals in The Netherlands have a statutory duty to report ‘critical incidents’ to the Dutch Health Care Inspectorate, defined as ‘an unintended or unexpected healthcare related event that resulted in the death or serious permanent injury to a patient [13].

### Study design and sample

The DHI database from 2008 to 2011 contained 357 ‘perinatal’ cases concerning various echelons of perinatal care reported by patients, midwives, general physicians, obstetricians, paediatricians and hospital boards. In the study described in this article, we excluded cases concerning women with a predefined high risk pregnancy profile and cases that were solely related to specialized neonatal care. We included all 89 reports in the database from January 2008 until December 2011 concerning care for women with an early low risk pregnancy profile, under supervision of a primary care midwife (and additional primary care providers). We also analysed the hospital care for these pregnant women in case of referral after 36 weeks. Further analysis focuses on the 71 reports which proved to be critical incidents as defined by the Dutch Quality Act. The other 18 reports were not specifically related to individual patient care or did not cause severe harm. Most of these remaining 18 reports were referred by the DHI to a committee for handling patients’ complaints. The content of the critical incident reports in the DHI database varied, but usually included parts of patient records, reports of interviews with patients and care providers, a variety of root cause analysis reports drawn by special safety committees in hospitals or the primary care providers themselves, and a final assessment by the DHI that focused on the possibility of repeated occurrence and the implementation of improvement measures in a specific hospital or practice.

### Ethical approval

The database of the DHI is accessible to researchers under the following three strict conditions; a signed confidentiality agreement, the information from the database is not identifiable to individual patients and, prior to publication, the DHI grants approval to the manuscript. Our study meets these conditions according to the ethics committee’s assessment.

### Measures

In an earlier national patient safety study in primary midwifery care we developed and validated an instrument for the review of records of healthy pregnant women to identify determinants of adverse outcomes and near misses [14]. This instrument is based on patient safety literature in general and obstetric and midwifery care in particular, and on clinical and managerial topics derived from practice guidelines. It reviews possible risk procedures and provides the classification of safety risk determinants in five risk areas where each case can contribute to one or more determinants: healthcare organization, communication about treatment, patient related risk factors, clinical management, and clinical outcomes. Although suboptimal clinical outcomes do not necessarily imply unsafe care, the care provided in these cases was perceived as requiring a detailed retrospective analysis.

The DHI performed a previous analysis of critical incidents in maternity care from 2006 to 2008. The case files were analysed to determine which factors contributed to the incidents, paying particular attention to care involving multiple caregivers, and care delivered after office hours. Actions and measures taken to prevent repeated occurrence were recorded [15]. The first mentioned instrument was developed for primary care. The DHI analysis also focused on hospital care. We complemented our instrument with specific DHI questions for primary and hospital care and aimed for an optimal detection of determinants and consequences of high- risk in both echelons of maternal care. (Additional file 1)

### Analysis

A multidisciplinary team of the DHI including perinatal and general DHI professionals analysed the incidents in the database and assessed the root cause analysis by care providers as well as the implementation of improvement measures to prevent recurrence in a specific hospital or primary care practice. A standardized overall analysis of determinants of risk in the cases that were reported from 2009 until 2011 has not been undertaken by the DHI.

For the analysis as described in this article, two trained reviewers – one academically trained research midwife and one DHI professional- independently analysed the incidents and the final DHI assessment using the above mentioned instrument. The reviewers were not allowed to request additional information because the cases were closed. Inconclusive results between reviewers were discussed with the two reviewers and with the DHI Inspector.

## Results

### General

#### Setting

Our study included 71 critical incidents: 42 (59%) incidents occurred in hospital care, 29 (41%) incidents happened in primary care. Eighteen incidents occurred in care provided by a midwife, six primary care incidents occurred when pregnant women in primary midwifery care consulted a general practitioner (GP). Five of these incidents were related to care in a GP out of office hours services. Four incidents in primary midwifery care were related to auxiliary care by a maternity assistant at home in the postnatal period and 1 incident was related to a public pharmacy.

#### Outcomes

In the 71 critical incidents were three cases of maternal death and eight critical incidents were recorded because of severe maternal morbidity. The records described 42 perinatal deaths and 12 critical incidents with severe morbidity for the child.

### Determinants of risk of the critical incident

We identified the determinants of risk as described in our instrument (Additional file 1) that contributed to a greater or lesser extent to the occurrence of the critical incident. Table 1 describes the potential determinants of the incidents.

**Table 1:** Classification of determinants of risk in the critical incidents

**Determinants of risk of the critical incident**	**Critical incidents (n = 71)**
**Availability of healthcare provider**	
Availability of the care provider in charge	20 (28%)
**Communication**	
Communication about treatment between care providers within a practice	39 (55%)
Communication about treatment between primary care and hospital caretakers	7 (10%)
Communication with the patient	7 (10%)
**Clinical management**	
Referral procedures	19 (27%)
Risk assessment by telephone triage	10 (14%)
Medication procedures	3 (4%)
Technical procedures	9 (13%)

#### Availability of healthcare provider

The timely availability of responsible care providers was assessed in relation to the timeframe of the urgent question for help, and that from the patient until arrival of the responsible care provider or availability of advice by telephone. In 20 cases there was a delay in the availability in primary or hospital care for more than 15 minutes in case of an urgent question for help.

#### Communication about treatment

Insufficient communication about treatment between caretakers within a primary practice or hospital was assessed as a potential cause in 39 cases. Communication between the primary and hospital care was a risk in seven incidents. In seven critical incidents communication problems with the patient were identified as a potential cause. These communication problems were described as ‘due to a language barrier’.

#### Clinical management

We analyzed 61 referrals during pregnancy (n = 35) and birth (n = 26). Twelve referrals during pregnancy from primary care to the hospital were delayed, and five women should have been referred according to practical guidelines but they were referred only after the critical incident occurred. Two women were referred during pregnancy from the hospital back to primary care but should have stayed in hospital care according to practice guidelines. Three referrals during birth were delayed. The remaining 39 referrals were timely and correct.

The risk assessment by telephone was a potential cause for safety risk in ten cases. In four out of these ten cases, the telephone triage was performed by a midwife, in five cases the triage was performed by a general physician service, and one case occurred in hospital care.

Incidents caused by medication (3) and technical procedures (7) were mainly described in cases that occurred after referral to hospital care.

#### Clinical management during out of office hours

In our analysis we found that 40 (56,3%) of the critical incidents occurred outside office hours and 31 (43,7%) during office hours. According to a retrospective assessment of the reviewers, 19 (26,8%) incidents may have had a better outcome if they had occurred during office hours. Six of these possibly avoidable out of office hours incidents occurred in primary care (n = 29). Three of these primary care incidents occurred in an out of office hours GP service and three in a midwifery practice. In two incidents in the GP service the GP nurse was not able to reach the responsible GP in time because of workload and in 1 incident the GP did not respond adequately to severe symptoms. In one incident in the midwifery practice the responsible midwife did not respond in time because of another urgent call for help, in one incident the midwife did not visit the patient at home in the late evening despite two calls for help and in 1 incident a colleague midwife from another practice was not available by telephone.

13 incidents during out of office hours occurred in a hospital. In seven incidents in hospital care a delay occurred in the availability of care providers such as the first or second obstetrician, pediatrician, and the surgery unit team. In four incidents there was a communication problem between the evening and night shift, in two of these cases the pregnant women were incorrectly referred back to primary care. In two incidents the responsible obstetrician was in the hospital during the night but the nurse or clinical midwife hesitated to call.

### Actions undertaken by the DHI

The DHI recommended and imposed one or more actions to prevent recurrence and supervise the implementation of such measures. This may vary from adjusting protocols on local or national level, to organizational adjustments or disciplinary actions. Table 2 describes the recommended actions according to the DHI.

**Table 2:** Measures imposed by the DHI to prevent recurrence

**Required improvement**	**N**
Diagnostic procedures and medical treatment	12
Organization of (urgent) care	26
Task description and delineation	14
Record keeping	20
Communication between care providers	24
Structural training	14
Written protocols	39
No measures recommended or recorded	11

In most cases the DHI imposed the improvement of written protocols followed by improving the organization of urgent care and better communication between care providers.

## Discussion

We performed a standardized analysis of critical incidents that were related to substandard care in primary midwifery care and subsequent hospital care for women with a low risk profile at the start of their pregnancy. We were able to identify a range of determinants that contributed to the occurrence of critical incidents.

In general, care for childbearing women is characterized by the possible need for urgent interventions [16]. Most professionals in perinatal care consider 15 minutes to be an acceptable maximum delay period to start urgent care in general [17]. Since the delayed availability of the care provider in charge was a potential cause of 20 (n = 71) critical incidents, this has to be considered. For instance, it has impact on the planning of geographical distribution of healthcare providers and the organisation of hospital care.

Studies have revealed that poor communication is a well-known risk for patient safety [18]. Given the substantial number of instances of miscommunication about treatment between care providers within primary care practices and within teams in maternity wards in hospitals, the first focus of improvement should be on the improvement of internal communication procedures by means of a standardized handover tool. Special attention is needed for Dutch language skills and translation since the existence of a language barrier is a crucial determinant of risk [19]. This can easily be improved by the use of an interpreter.

In patient safety literature, technical failure such as emergency calls and medication errors are well known for their consequences for patient safety [20]. Our evaluation also showed that these factors contribute to the occurrence of critical incidents and are therefore in need of test procedures of emergency call systems and medication safety programs.

Timely referral contributes to safe care for pregnant women. There is a low prevalence of severe pregnancy related problems in primary care and the presentation of these complications by pregnant women can be difficult to interpret. Despite a perceived low risk of harm in primary care, a continuous awareness of a possible presentation of high risk complications especially during pregnancy, should be part of daily practice.

Five out of nine telephone triage related incidents happened in a GP out of office service. Although primary care midwives and maternity wards in hospitals offer 24/7 care, pregnant women also call the out of office GP services with general or pregnancy related problems. A study on the safety in telephone triage in out- of- hours care shows that there’s room for improvement of triage in patients who present high risk symptoms [21]. This emphasizes the importance of knowledge about the current state of clinical management and guidelines in care for pregnant women and, in case of doubt, consultation from the GP with the responsible maternity care provider. In addition, pregnant women should be informed to contact their primarily responsible care provider in case of pregnancy related problems.

### Limitations

In our earlier research in primary midwifery care, we described the underestimation of the level of risk on the basis of the medical or obstetric risk (e.g. small for gestational age child in the obstetric history) and lifestyle factors associated with safety incidents [10]. In our current analysis it was not possible to review these factors, since there was no structural notation in the previous root cause analysis by care providers. Further, additional information, such as birth weight, gestational age, and information from the records of primary care in case of referral to a hospital, was not structurally presented by care providers. Since we were not authorized to request for additional information, our current analysis and the description of the incidents in this article were thus restricted to the reports and data that were available in the database. It is difficult to draw firm conclusions about causality and a possible correlation between safety determinants from a case-by-case analysis but our structural approach provided us with some safety highlights.

## Conclusion

We reviewed the critical incidents and the DHI assessment of cases in primary and subsequent hospital care for pregnant women with a low risk profile in early pregnancy. We performed an analysis of cases that were reported to the DHI and that did not reflect the population at large. Since all cases had an unexpected or unintended care related component, we were able to identify determinants of risk that contributed to the occurrence of the incident. Our standardized analysis provides additional and more valid information compared to the non-systematic evaluation that is currently performed by involved care providers or by the DHI. We used a standardized instrument and aimed for the detection of determinants of high risk. The variety of potential determinants and improvement measures that are recommended, substantiate the conclusion that no easy solutions for patient safety questions exist.

Over the last few years, perinatal care providers implemented a successful perinatal audit system in The Netherlands. Also the DHI recommends improvement measures and supervises the quality and safety of healthcare. Our study shows that the structural implementation of a standardized analysis of unintended or unexpected care related events can provide valuable information to all health care providers and improve the awareness towards the influence of safety determinants on the occurrence of a critical incident. Our analysis strengthens the importance of routine data collection on the determinants of safety risks as described in the instrument.

The majority of pregnant women has a low risk profile in early pregnancy and are cared for by primary care midwives. These women are frequently referred during pregnancy and birth and therefor it’s important to analyse the complete spectrum of primary and hospital care for these women. Because of a low a priori chance of adverse outcomes in low risk pregnancies, a special focus on critical incidents in this population provides all primary care midwives with valuable information, regardless their involvement in a case. Given the increased willingness to report critical incidents throughout the last years, an analysis of these incidents and a structural report of the findings to primary care midwives, will contribute to the awareness of safety risks and improve the quality of care.

## Competing interests

The authors declare that they have no competing interests.

## Authors’ contributions

LM and RW performed the analysis of the database. MA supervised the analysis. LM, MA and AJ developed the study instrument. LM drafted the manuscript with help from SB and MW. All authors were involved in the interpretation of the data and writing the manuscript. All authors read and approved the final manuscript.

## Pre-publication history

The pre-publication history for this paper can be accessed here:

http://www.biomedcentral.com/1471-2393/13/219/prepub

## Supplementary Material

Additional file 1 Instrument for the review of potential determinants of safety risks. Click here for file
