# Supplementary material for: Adverse outcomes in maternity care for women with a low risk profile in The Netherlands: a case series analysis
Source: BMC Pregnancy Childbirth. 2013 Nov 29;13:219. doi: 10.1186/1471-2393-13-219 (PMC4219453; doi:10.1186/1471-2393-13-219)
Supplement: Additional file 1 — Instrument for the review of potential determinants of safety risks. [file 1471-2393-13-219-S1.doc]

**Additional file 1. Appendix** Instrument for the review of potential determinants of safety risks

| **Determinants of safety risk** |
| --- |
| **Organization**  delay in arrival/ availability by telephone of responsible care provider (more than 15 minutes)  Delay in attainability of hospital care (more than 45 minutes)  Delay in ambulance transportation to hospital (more than 45 minutes)  Birth with no responsible care provider present (birth before arrival) |
| **Communication**  Communication incident with care providers (inside practice)  Communication incident with other care providers (outside practice)  Communication incident with patient |
| **Patient risk factors**  Presence of general risk factors  Presence of social risk factors  Presence of lifestyle factors  Presence of mental risk factors  Use of medication  Presence of risk factors in family history  Presence of risk factors in obstetric history  Woman does not follow prescribed therapy and no show |
| **Clinical management**  Incidents during preventive procedures  Incidents during diagnostic procedures  Medication incidents  Technical failure  Number of contacts, first antenatal visit after 10 weeks pregnancy or < 12 consults in full antenatal care  Incidents in referral procedures in this pregnancy  Incidents in referral procedures during/after birth  Incidents in risk assessment by telephonic triage |
| **Outcomes**  *Neonatal outcome:*  small or large for gestational Age, low Apgar score <7 after 5 minutes, breech delivery, congenital abnormalities, birth trauma, hospital admission of the child, severe morbidity, mortality.  *Maternal outcome:*  anemia, complicated instrumental delivery or caesarean section, prolonged hospitalization, inadequate coping after postnatal period, traumatic experience of birth, suspicion of depression or psychosis, severe morbidity, Mortality |
| **DHI**  Investigation by the DHI  Quality of recordkeeping  Records available from primary care and hospital care  Care during out of office hours  Problems with ‘chain care’ between primary care and hospital care  Measures to prevent recurrence |
